# Sulforaphane prevents LPS-induced inflammation by regulating the Nrf2-mediated autophagy pathway in goat mammary epithelial cells and a mouse model of mastitis

**DOI:** 10.1186/s40104-023-00858-9

**Published:** 2023-05-03

**Authors:** Dan Shao, Wenxiang Shen, Yuyang Miao, Zhen Gao, Menghao Pan, Qiang Wei, Zuoting Yan, Xiaoe Zhao, Baohua Ma

**Affiliations:** 1 Key Laboratory of Animal Biotechnology of the Ministry of Agriculture, College of Veterinary Medicine, Northwest A&F University, Yangling, 712100 Shaanxi China; 2 Lanzhou Institute of Husbandry and Pharmaceutical Sciences, Chinese Academy of Agricultural Science, Lanzhou, 730050 China

**Keywords:** Autophagy, Goat mammary epithelial cells, Inflammation, Nrf2, Oxidative stress, Sulforaphane

## Abstract

**Background:**

Mastitis not only deteriorates the composition or quality of milk, but also damages the health and productivity of dairy goats. Sulforaphane (SFN) is a phytochemical isothiocyanate compound with various pharmacological effects such as anti-oxidant and anti-inflammatory. However, the effect of SFN on mastitis has yet to be elucidated. This study aimed to explore the anti-oxidant and anti-inflammatory effects and potential molecular mechanisms of SFN in lipopolysaccharide (LPS)-induced primary goat mammary epithelial cells (GMECs) and a mouse model of mastitis.

**Results:**

In vitro, SFN downregulated the mRNA expression of inflammatory factors (tumor necrosis factor-α (*TNF-α*), interleukin (*IL*)-*1β* and *IL-6*), inhibited the protein expression of inflammatory mediators (cyclooxygenase-2 (COX2), and inducible nitric oxide synthase (iNOS)) while suppressing nuclear factor kappa-B (NF-κB) activation in LPS-induced GMECs. Additionally, SFN exhibited an antioxidant effect by increasing Nrf2 expression and nuclear translocation, up-regulating antioxidant enzymes expression, and decreasing LPS-induced reactive oxygen species (ROS) production in GMECs. Furthermore, SFN pretreatment promoted the autophagy pathway, which was dependent on the increased Nrf2 level, and contributed significantly to the improved LPS-induced oxidative stress and inflammatory response. In vivo, SFN effectively alleviated histopathological lesions, suppressed the expression of inflammatory factors, enhanced immunohistochemistry staining of Nrf2, and amplified of LC3 puncta LPS-induced mastitis in mice. Mechanically, the in vitro and in vivo study showed that the anti-inflammatory and anti-oxidative stress effects of SFN were mediated by the Nrf2-mediated autophagy pathway in GMECs and a mouse model of mastitis.

**Conclusions:**

These results indicate that the natural compound SFN has a preventive effect on LPS-induced inflammation through by regulating the Nrf2-mediated autophagy pathway in primary goat mammary epithelial cells and a mouse model of mastitis, which may improve prevention strategies for mastitis in dairy goats.

## Introduction

Mastitis, an infection, and inflammation of the mammary gland, is one of the most prevalent diseases of dairy animals during the lactation period [1]. Not only does it affect the nutritional value or composition of milk, but it also affects animal health and hygienic quality, causing huge economic losses associated with dairy products [2]. Emerging evidence has indicated that inflammatory diseases are accompanied by oxidative stress initiated by the accumulation of reactive oxygen species (ROS) [3, 4]. Considering this, it is reasonable to suggest that combined regulation of inflammatory response and oxidative stress, rather than a specific therapy targeting one of these systems [5], would be more successful in controlling the progression of mastitis. Therefore, it is highly necessary to develop a safe agent for preventing mastitis with retained anti-inflammatory and anti-oxidative stress functions.

Nrf2 is a key transcription factor that regulates the expression of the antioxidant proteins such as heme oxygenase-1 (HO-1), NADPH quinone oxidoreductase 1 (NQO1), glutamate-cysteine ligase catalytic (GCLC), and glutamate cysteine ligase modifier subunit (GCLM), by binding to the antioxidant response element (ARE) [6, 7]. This pathway is essential to the cellular antioxidant and anti-inflammatory defense systems [8–10]. Indeed, the upregulation of Nrf2 increases the expression of antioxidant protein for ROS scavenging [11, 12]. Additionally, Nrf2 can compete with NF-κBp65 for interacting with p300/CREB binding protein (CBP), thereby reducing NF-κB-driven gene expression [13, 14]. Previous studies have demonstrated that Nrf2 participates in mediating lipopolysaccharide (LPS)-induced mice mastitis [12, 15, 16]. Consequently, Nrf2 is a potential marker associated with mastitis resistance.

Autophagy is an evolutionarily conserved self-renewal process, which delivers cytoplasmic constituents, such as misfolded proteins, damaged organelles, and invading pathogens, into autophagosomes and subsequently transports them to the lysosomes for degradation [17, 18]. This process is critical for maintaining cellular homeostasis and regulating the host’s response to infection by managing oxidative stress and inflammation [19–21]. For instance, transcription factor EB (TFEB)-mediated autophagy alleviates oxidative damage in bovine mammary epithelial cells [22]. Moreover, increasing evidence indicates that autophagy participates in mediating LPS-induced inflammation [15, 16, 23]. It has been proposed that Nrf2 enhances the expression of several autophagy-related genes and promotes the autophagic process through binding to its ARE site [24, 25]. Therefore, it is clear that autophagy and the Nrf2 pathway are intrinsically intertwined, suggesting that the activation of Nrf2-mediated autophagy might be a novel and feasible approach for the treatment of inflammation research [26, 27].

Many natural compounds in food may be considered the oldest medicines due to their bioactive ingredients, commonly known as nutraceuticals, which possess various physiological and pharmacological properties with limited side effects [28–30]. Sulforaphane (SFN), a natural electrophilic compound, isolated from cruciferous vegetables such as broccoli and cabbage [31], is one such compound. Numerous studies have reported that SFN has a potential role in the treatment of diseases induced by oxidative stress and inflammatory response, as it promotes promoting the nuclear accumulation of Nrf2 [32–34]. Despite being well characterized for its role in regulating mastitis, little is known about its potential mechanisms. Therefore, the present study aimed to investigate the anti-oxidant and anti-inflammatory effects and potential molecular mechanisms of SFN in LPS-induced primary GMECs and a mouse model of mastitis.

## Materials and methods

### Regents

Sulforaphane (purity > 99%, HY-13755) and chloroquine (CQ, HY-17589A) were obtained from MedChemExpress (Shanghai, China). LPS (L2880) was bought from Sigma Aldrich (Shanghai, China). Bafilomycin A1 (Baf-A1, S1413) was purchased from Selleck Chemicals (Houston, TX, USA).

### Ethics statement

All animal experiments for the study were approved by the Institutional Animal Care and Use Committee of Northwest A&F University (No.2021032610).

### Mice and treatments

BALB/c mice (8–9 weeks old, 20–25 g weight) were purchased from the Laboratory Animal Center of Shaanxi Normal University (Xian, China) and were maintained under a standard environment (12 h light/dark cycle, 22 – 23 ℃) with ad libitum access to food and water. Two females and one male were distributed in a small cage. After pregnancy, each female mouse was housed individually and subjected to different treatments. A total of forty lactating mice were randomly divided into four groups (*n* = 10 per group): Control, LPS (0.2 mg/mL, 50 µL), SFN (50 mg/kg/d), and LPS + SFN (50 mg/kg/d). The dosages of SFN and LPS in the animal models were based on the literature [33, 35]. Mice were administered intraperitoneally with SFN for 7 d before LPS treatment from days 4 to 10 of lactation. On day 9 of lactation, LPS was injected into the fourth mammary duct of the mice using a microsyringe. Twenty-four hours after the LPS injection, all animals were sacrificed, and mammary gland samples were collected.

### GMEC isolation and culture

Goat mammary epithelial cells were isolated from three healthy lactating dairy goats using the tissue explant culture method as previously described [36]. Briefly, the mammary tissue blocks were washed 5 – 6 times with phosphate-buffered solution (PBS) and cut into 1mm^3^ pieces, the pieces were then transferred to 24-well plates and incubated in a humidified atmosphere containing 5% CO_2_ at 37 °C. After 12 h, Dulbecco Modified Eagle Medium/nutrient mixture Ham F-12 (DMEM/F12, 12500, Thermo Fisher Scientific, Waltham, MA, USA) supplemented with 10% fetal bovine serum (FBS; Thermo Fisher Scientific, 10270106), 100 U/mL penicillin, and 100 µg/mL streptomycin (Solarbio, Beijing, P1400) was added into the culture plates. The medium was replaced with fresh medium every 48 h until the cells crawled out of the tissue. After about 10 d, the cells distributed across the bottom of the plates were digested with 0.25% trypsin–EDTA to remove fibroblasts. Following three passages of purification, the isolated GMECs were used for subsequent experiments within seven passages.

### Cell toxicity assay

The cytotoxicity of primary GMECs in response to SFN was evaluated using Cell Counting Kit-8 (CCK-8, AbMole Bioscience Inc., Houston, TX, USA). Briefly, GMECs were seeded in 96-well plates at a density of 8 × 10^3^ cells/well and cultured with various concentrations of SFN for 24 h. Then, 10 µL of CCK-8 reagent and 90 µL of culture medium were added to each well and incubated for 2 h at 37 °C. Subsequently, the absorbance of the samples at 450 nm was measured.

### Quantitative real-time PCR analysis

Total RNA was isolated using AG RNAex Pro Reagent (Accurate Biology, Changsha, China) and transcribed into cDNA using the Evo M-MLV Kit (Accurate Biology, Changsha, China). Quantitative real-time PCR (qRT-PCR) analysis was carried out using SYBR® Green Pro Taq HS (Accurate Biology, Changsha, China) under the following conditions: 95 ℃ for 30 s; 40 cycles of 95 ℃ for 5 s and 60 ℃ for 30 s, with final melting-curve analysis. The primer sequences used are listed in Table 1. Relative gene expression was calculated by normalizing the housekeeping gene *β-actin* in mouse mammary gland tissue or *GAPDH* in primary GMECs using the comparative 2^-^^∆∆Ct^ method.

**Table 1:** Primers sequences for qRT-PCR

Gene	Sequence (5′ → 3′)	Length, bp
Goat *Nrf2*	F: CCAACTACTCCCAGGTAGCCC R: AGCAGTGGCAACCTGAACG	227
Goat *HO-1*	F: CAAGCGCTATGTTCAGCGAC R: GCTTGAACTTGGTGGCACTG	206
Goat *NQO1*	F: ACTGTGTCGGACCTGTATGC R: CAGAGAGTACATGGAGCCGC	363
Goat *GCLM*	F: AATCTTGCCTCCTGCTGTGTGATG R: GATGCTCTCCTGAAGTGCTTCTTGG	138
Goat *GCLC*	F: CATTTGCAAAGGTGGCAACGC R: CTGCTTGTAGTCGGGATGCT	301
Goat *GAPDH*	F: ACCTGCCAAGTATGATGAG R: AGTGTCGCTGTTGAAGTC	118
Mouse *TNF-α*	F: ACGGCATGGATCTCAAAGAC R: GTGGGTGAGGAGCACGTAGT	116
Mouse *IL-1β*	F: GCTGCTTCCAAACCTTTGAC R: AGCTTCTCCACAGCCACAAT	121
Mouse *IL-6*	F: CCGGAGAGGAGACTTCACAG R: CAGAATTGCCATTGCACAAC	134
Mouse *β-actin*	F: GTCAGGTCATCACTATCGGCAAT R: AGAGGTCTTTACGGATGTCAACGT	147

### Western blot analysis

Western blot was performed as previously described [37]. Briefly, total protein was extracted from GMECs in ice-cold RIPA buffer (Solarbio, Beijing, China) with a protease and phosphatase inhibitor cocktail (Solarbio, Beijing, China). Protein concentration was measured using a bicinchonic acid (BCA, YuanYe, Shanghai, China) protein assay kit (Epizyme, Shanghai, China). Equal amounts of protein were separated by sodium dodecyl sulfate–polyacrylamide gel electrophoresis (SDS-PAGE) and then transferred onto polyvinylidene fluoride membranes (Millipore, Bedford, MA, USA). Primary antibodies were incubated overnight at 4 °C followed by a 2-h incubation with secondary antibodies at room temperature. Band intensities were visualized and determined using a chemiluminescence detection system (Bio-Rad Laboratories, Hercules, CA, USA). The intensity of each band was analyzed using Image J. Primary antibodies used were: IκBα (1:1,000, #4814, Cell Signaling Technology, Boston, MA, USA), p-IκBα (1:1,000, #2859, Cell Signaling Technology), NF-κB p65 (1:1,000, 10745–1-AP, Proteintech, Wuhan, China), p-NF-κB p65 (1:1,000, #3033, Cell Signaling Technology), Nrf2 (1:2,000, ab137550, Abcam, Cambridge, USA), HO-1 (1:2,000, 10701–1-AP, Proteintech), NQO1 (1:2,000, 11451–1-AP, Proteintech), GCLC (1:2,000, 12601–1-AP, Proteintech), GCLM (1:2,000, 14241–1-AP, Proteintech), LC3 (1:1,000, 14600–1-AP, Proteintech), Beclin1 (1:1,000, 11306–1-AP, Proteintech), Atg7 (1:1,000, 10088–2-AP, Proteintech), ULK1 (1:1,000, 20986–1-AP, Proteintech), p-ULK1S556 (1:2,000, 80218–1-RR, Proteintech), p-ULK1S757 (1:1,000, #14202, Cell Signaling Technology), AMPK (1:1,000, #5831, Cell Signaling Technology), p-AMPK (1:1,000, #2535, Cell Signaling Technology), mTOR (1:1,000, #2983, Cell Signaling Technology), p-mTOR (1:1,000, #5536, Cell Signaling Technology), S6 (1:1,000, #2217, Cell Signaling Technology), and p-S6 (1:2,000, #4858, Cell Signaling Technology).

### ROS analysis

The ROS levels were then measured using DCFH-DA fluorescent dye (Beyotime Institute of Biotechnology, Shanghai, China). Briefly, GMECs were cultured in 24-well plates and subjected to indicated treatments. Then cells were incubated with DCFH-DA (10 μmol/L) for 20 min at 37 °C in the dark. The cells were washed with PBS and the fluorescence was imaged using a fluorescence microscope (Olympus, Tokyo, Japan). The fluorescence intensity was quantified using Image J software (1.53a/Java 1.8.0_112, NIH, USA).

### RNA interference (RNAi) of Nrf2

The small interfering RNA (siRNA, GenePharma, Shanghai, China) targeting Nrf2 and negative control RNA (NC siRNA) were designed and synthesized by GenePharma (Shanghai, China). At 70% – 80% confluence, GMECs were transfected with siRNA (50 nmol/L) using Lipofectamine 3000 (Invitrogen, Carlsbad, CA, USA). After 48 h, transfected cells were used for subsequent experiments were performed. The Oligos for siRNA are listed in Table 2.

**Table 2:** Oligos for siRNA

Gene	Sequence (5′ → 3′)
si-NC	UUCUCCGAACGUGUCACGUTT ACGUGACACGUUCGGAGAATT
si-Nrf2-1	GAGGCCAGAUAUUAAGAAATT UUUCUUAAUAUCUGGCCUCTT
si-Nrf2-2	CCGGUUGACAGUGAAUUCATT UGAAUUCACUGUCAACCGGTT
si-Nrf2-3	GGUAGCCACUGCUGAUUUATT UAAAUCAGCAGUGGCUACCTT

### Hematoxylin–eosin staining

Mammary gland histology was performed as follows. In brief, the tissue was fixed in 4% paraformaldehyde, embedded in paraffin, and sectioned to a thickness of 5-μm. Mammary sections were then stained with hematoxylin–eosin to evaluate pathological changes.

### Immunohistochemistry

Immunohistochemistry (IHC) was performed on paraffin-embedded udder tissue sections using a previously described protocol [33]. An anti-Nrf2 primary antibody was used (1:400; ab137550, Abcam, Cambridge, USA).

### Immunofluorescent staining

GMECs were grown on glass coverslips and then fixed with 4% paraformaldehyde for Nrf2 and NF-κB p65 staining or −20 °C methanol for LC3 staining. Following this, cells were permeabilized with 0.1% Triton X-100 and then blocked with 10% FBS in PBS for 1 h. Cells were then incubated overnight with different primary antibodies at 4 °C. The primary antibodies used in this study included anti-NFE2L2/Nrf2 (1:400, ab137550, Abcam, Cambridge, USA), anti-NF-κB p65 (1:200, 10745–1-AP, Proteintech, Wuhan, China) and LC3 (1:300, 14600–1-AP, Proteintech). Cells were then washed five times with PBS and incubated with secondary antibodies conjugated to Alexa Fluor 594 (A-11012, Thermo Fisher Scientific, Waltham, MA, USA) or 488 (A-11008, Thermo Fisher Scientific) for 1 h in a dark room at room temperature. After three washes with PBS, coverslips were mounted on slides with Antifade Mounting Medium (Beyotime), and images were acquired using a confocal microscope (Nikon A1Rsi, Nikon, Tokyo, Japan).

For tissue immunofluorescence, paraffin-embedded mouse breast sections were deparaffinized for subsequent antigen retrieval. Subsequently, udder sections were blocked with goat serum and incubated with a primary antibody against LC3 (1:300; 14600–1-AP, Proteintech) at 4 °C overnight, followed by staining with fluorescein isothiocyanate (FITC)-conjugated donkey anti-rabbit secondary antibody (1:200; GB22403, Servicebio, Wuhan, China). Slides were counterstained with DAPI, and images were captured using a fluorescence microscope (Nikon, Tokyo, Japan).

### Transmission electron microscopy

Cells were harvested and prefixed in 2.5% glutaraldehyde followed by postfixation in 1% osmium tetroxide. After dehydration, embedding, and ultrathin sectioning, the samples were stained with uranyl acetate and lead citrate and then imaged using a JEM-1400-FLASH transmission electron microscope (Tokyo, Japan).

### Statistical analysis

The values are expressed as mean ± SEM. Statistical analysis was performed using GraphPad Prism 8.0.1 software (GraphPad, San Diego, CA, USA). Student’s *t*-test was performed for comparisons between the two groups and one- or two-way analysis of variance (ANOVA) followed by Tukey or Dunnett’s post-hoc tests were used to compare more than two groups. Statistical significance was denoted by asterisks (^*^P < 0.05, ^**^P < 0.01, ^***^P < 0.001, ^****^P < 0.0001).

## Results

### SFN inhibits inflammatory factors, inflammatory mediators expression, and NF-κB (p65) activation in LPS-induced primary GMECs

**Fig. 1:**
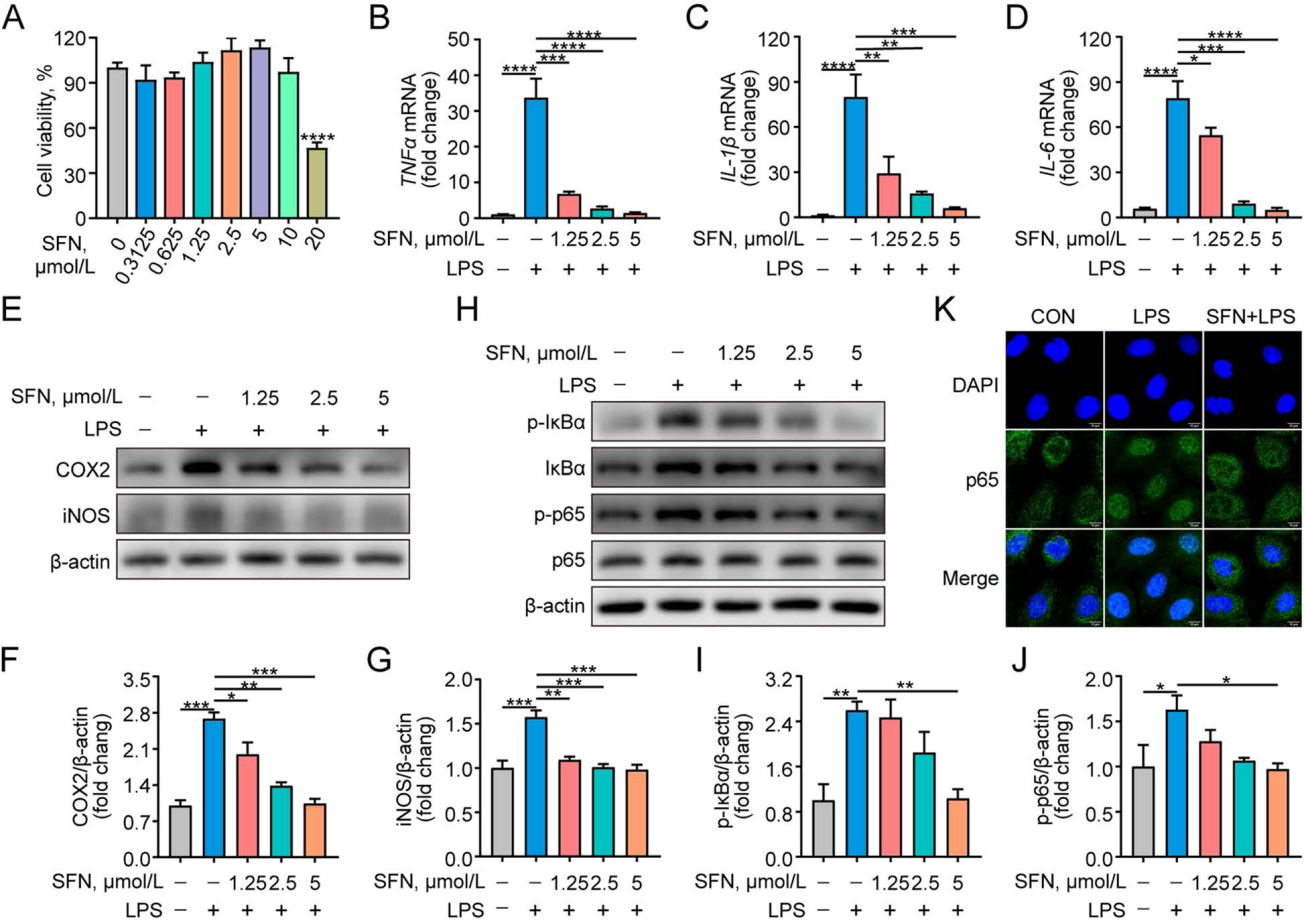
SFN inhibited expression of inflammatory factors and mediators and NF-κB (p65) activation in LPS-induced primary GMECs. Cells were pretreated with different concentrations of SFN (1.25, 2.5 and 5 μmol/L) for 12 h in the absence or presence of LPS (10 μg/mL) for 6 h. **A** GMECs were treated SFN for 24 h and the cytotoxicity of SFN was detected using CCK-8 assay (*n* = 4). **B–D** qRT-PCR analysis of relative mRNA levels of inflammatory factors (*n* = 3). **E–G** Western blot analysis of COX2 and iNOS levels (*n* = 3). **H–J** Western blot analysis of p-IκBα, IκBα, p-p65 and p65 (*n* = 3). **K** Immunofluorescent analysis of NF-κBp65 and nuclear staining with DAPI (blue) in GMECs pretreated with SFN (5 μmol/L) for 12 h in the absence or presence of LPS (10 μg/mL) for 6 h (Bar = 10 μm). One-way ANOVA, Dunnett's post-hoc test. Data are presented as the mean ± SEM. ^*^P < 0.05, ^**^P < 0.01, ^***^P < 0.001 and ^****^P < 0.0001

As detected by the cytotoxicity assay, SFN within 10 μmol/L did not affect the cell viability of GMEC (*P* > 0.05). As depicted in Fig. 1B–D, the *TNF-α*, *IL-1β*, and *IL-6* mRNA levels were up-regulated in LPS-induced GMECs compared to the control group (*P* < 0.0001), and this effect was dose-dependently suppressed with SFN pretreatment (*P* < 0.05). Similarly, SFN inhibited LPS-induced protein expression of inflammatory mediators (COX2 and iNOS) (Fig. 1E–G, *P*< 0.05). Given that NF-κB is a major factor in regulating the expression of inflammatory cytokines, the effect of SFN on LPS-induced NF-κB pathway activation was further explored in primary GMECs. Western blot showed that SFN down-regulated LPS-induced phosphorylation levels of IκBα and NF-κB p65 proteins (Fig. 1H, *P* < 0.05). Furthermore, immunofluorescence analysis revealed that SFN pretreatment reduced the amount of p65 in the nucleus of LPS-induced GMECs (Fig. 1K). These data suggest the anti-inflammation effects of SFN on LPS-induced GMECs.

### SFN suppresses ROS production, up-regulates antioxidant enzymes expression, and activates Nrf2 signaling in LPS-induced GMECs

**Fig. 2:**
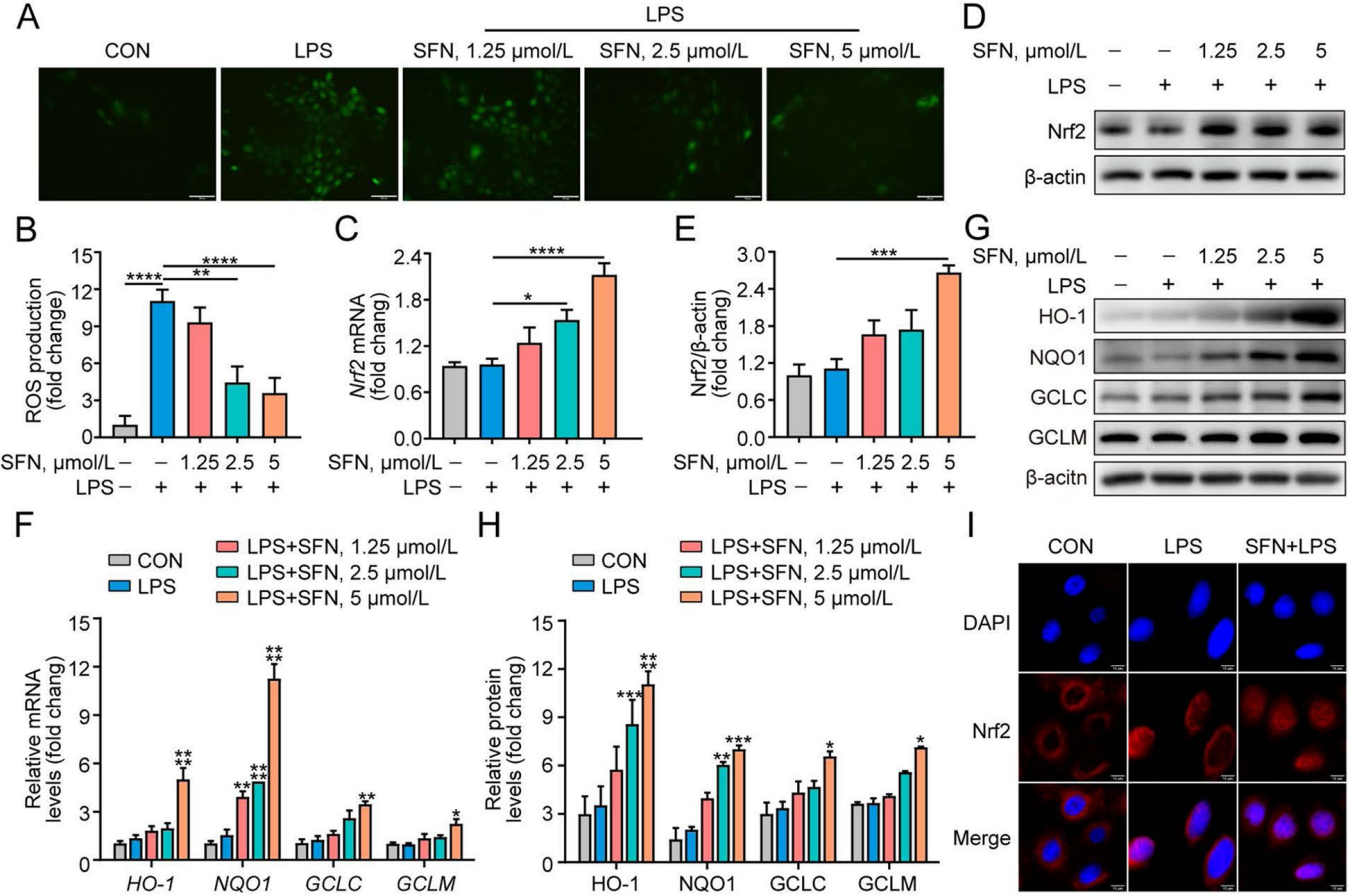
SFN suppressed ROS production, up-regulated expression of antioxidant enzymes and activated Nrf2 signaling in LPS-induced GMECs. Cells were pretreated with different concentrations of SFN (1.25, 2.5 and 5 μmol/L) for 12 h in the absence or presence of LPS (10 μg/mL) for 6 h. **A** ROS production were detected by DCFH-DA (Bar = 100 μm).** B** Quantification of ROS levels. One-way ANOVA, Dunnett's post-hoc test (*n* = 6). **C** qRT-PCR analysis of *Nrf2* mRNA (*n* = 3). One-way ANOVA, Dunnett's post-hoc test. **D** and **E** Western blot analysis of Nrf2 protein (*n* = 3). One-way ANOVA, Dunnett's post-hoc test. **F** qRT-PCR analysis of Nrf2-targeted genes *HO-1*, *NQO1*, *GCLC* and *GCLM* mRNA expression (*n* = 3). Two-way ANOVA, Dunnett's post-hoc test. **G** and **H** Western blot analysis of Nrf2 downstream proteins HO-1, NQO1, GCLC and GCLM (*n* = 3) levels. Two-way ANOVA, Dunnett's post-hoc test. **I** Immunofluorescent analysis of Nrf2 and nuclear staining with DAPI (blue) in GMECs pretreated with SFN (5 μmol/L) for 12 h in the absence or presence of LPS (10 μg/mL) for 6 h (Bar = 10 μm). Data are presented as the mean ± SEM. ^*^P < 0.05, ^**^P < 0.01, ^***^P < 0.001 and ^****^P < 0.0001

LPS has been shown to induce the accumulation of ROS in RAW264.7 cells [5], and ROS plays a crucial role in the inflammatory signaling pathway. Therefore, the impact of SFN on ROS generation was examined in LPS-induced GMECs by DCFH-DA assay. As depicted in Fig. 2A and B, ROS production significantly increased after LPS exposure compared to the control group (*P* < 0.0001). However, SFN pretreatment notably restrained LPS-induced ROS accumulation in a dose-dependent manner (*P* < 0.01). Nrf2 can activate the transcription of several antioxidant genes and has been proposed to be relevant to anti-inflammatory function in bovine MECs [15]. Therefore, we evaluated the effects of SFN on the expression of Nrf2 in LPS-induced GMECs. qRT-PCR and Western blot analysis revealed that SFN up-regulated Nrf2 gene and protein expression in the presence of LPS (Fig. 2C–E, *P* < 0.05). Furthermore, immunofluorescence staining showed that SFN increased nuclear accumulation of Nrf2 in LPS-induced GMECs (Fig. 2I). At the same time, the expression of phase II detoxifying enzymes including HO-1, NQO1, GCLC, and GCLM, which are known to alleviate oxidative stress and inflammatory response [38], was potentiated by SFN upon LPS treatment (Fig. 2F–H, *P* < 0.05). These results verify that the SFN-induced suppression of oxidative stress is related to Nrf2 activation and the expression of antioxidant enzymes.

### SFN-induced autophagy plays an important role in LPS-induced primary GMECs

**Fig. 3:**
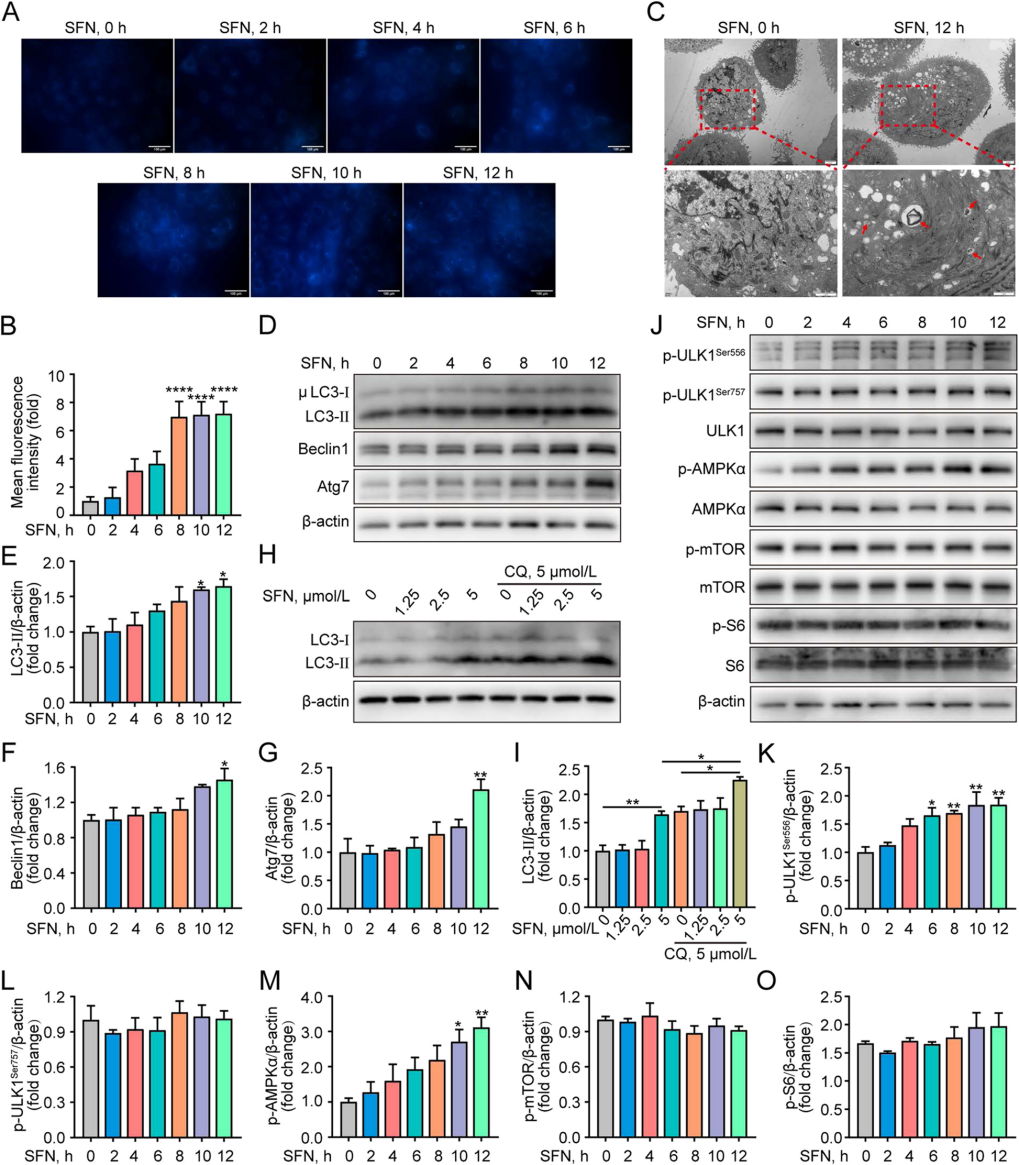
SFN promoted autophagy in primary GMECs. **A** GMECs were treated with SFN (5 μmol/L) for the indicated times and the autophagosome was detected using the autophagy assay kit (Bar = 100 μm). **B** Fluorescence intensity analysis of autophagy shown in **A**, *n* = 30 randomly selected cells. One-way ANOVA, Dunnett's post-hoc test. **C** Autophagosome detection by transmission electron microscopy in GMECs treated with SFN (5 μmol/L) for 12 h. Arrows indicate the autophagosome. **D–G** Western blot analysis of LC3, Beclin1, Atg7 protein from 5 μmol/L SFN-treated GMECs for the indicated times (*n* = 3). One-way ANOVA, Dunnett's post-hoc test. **H** and **I** Western blot analysis of LC3 protein (*n* = 3). GMECs were pretreated with or without CQ (5 μmol/L) for 4 h in advance and then were incubated with SFN (1.25, 2.5 and 5 μmol/L) for 12 h. One-way ANOVA, Tukey's post-hoc test. **J–O** Western blot analysis of the autophagy-related signaling molecules from 5 μmol/L SFN-treated GMECs for the indicated times (*n* = 3). One-way ANOVA, Dunnett's post-hoc test. Data are presented as the mean ± SEM. ^*^P < 0.05, ^**^P < 0.01, ^***^P < 0.001 and ^****^P < 0.0001

To examine the optimal times of SFN coordinates autophagy, primary GMECs were incubated with SFN (5 μmol/L) at different times. Autophagy was assessed using a kit-based assay, which revealed that SFN activated the typical autophagic response characterized by bright blue dot staining of autophagic vacuoles. The effect is noticeable after 4–12 h of treatment, with peak activity at 12 h (Fig. 3A and B, *P* < 0.0001). Furthermore, transmission electron microscopy images showed predominant autophagosome formation in SFN-treated GMECs (5 μmol/L, 12 h) (Fig. 3C). In support of this, Western blot analysis showed that SFN (5 μmol/L, 12 h) treatment up-regulated autophagy markers such as LC3-II, Beclin1, and Atg7 expression in GMECs (Fig. 3D–G, *P* < 0.05). To examine whether SFN promotes autophagic flux, CQ, a later-phase autophagy inhibitor, was used to treat GMECs combination with SFN. The results showed that SFN (5 μmol/L, 12 h) further increased LC3-II expression in GMECs after CQ treatment (Fig. 3H and I, *P* < 0.05). Additionally, the related pathways of autophagy were detected. It was observed that the phosphorylation level of AMPK, a major regulatory pathway of autophagy, was significantly elevated (Fig. 3J and M, *P* < 0.05), whereas the activity of mTOR and its downstream signaling molecule phos-S6 were not changed after SFN (5 μmol/L, 12 h) treatment (Fig. 3J, N and O, *P* > 0.05). Notably, SFN treatment also elevated the phosphorylation level of ULK1 at Ser556 (Fig. 3J and K, *P* < 0.05), but not Ser757 (Fig. 3J and L, *P* > 0.05). Taken together, these results demonstrate that SFN (5 μmol/L, 12 h) induces autophagy and promotes autophagosome formation, which is possibly dependent on AMPK-regulated ULK1 phosphorylation at Ser556.

**Fig. 4:**
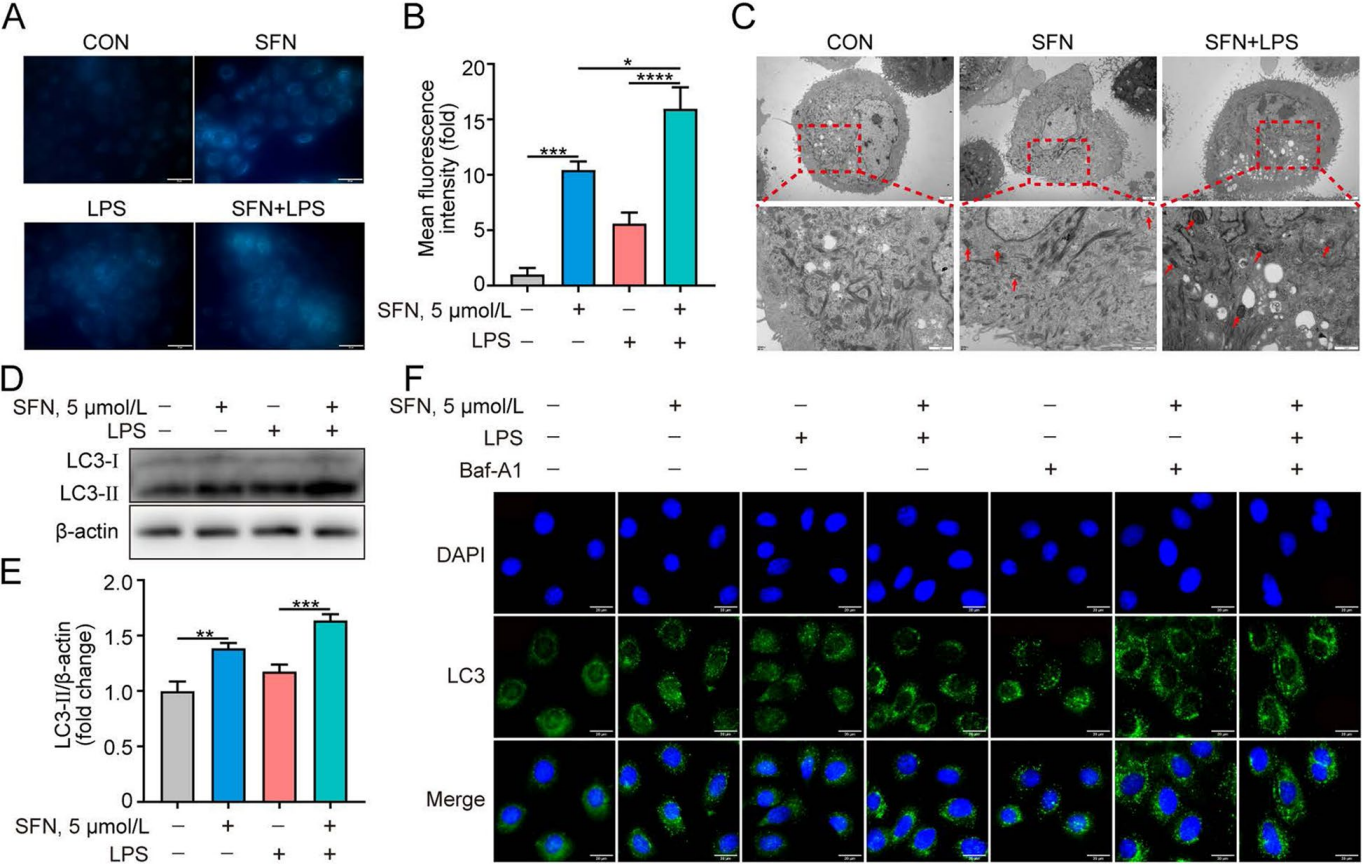
Autophagy activation by SFN was critical in LPS-induced primary GMECs. Cells were pretreated with SFN (5 μmol/L) for 12 h in the absence or presence of LPS (10 μg/mL) for 6 h. **A** Autophagy assay kit was used to detect autophagosome (Bar = 50 μm). **B** Fluorescence intensity analysis of autophagy shown in **A**, *n* = 30 randomly selected cells. **C** Autophagosome detection by transmission electron microscopy. Arrows indicate the autophagosome. **D** and **E** Western blot analysis of LC3 protein (*n* = 3). **F** Immunofluorescent analysis of LC3 puncta (green) and nuclear staining with DAPI (blue) in GMECs pretreated with Baf-A1 (10 μmol/L) for 1 h in advance and then treated with SFN (5 μmol/L) for 12 h in the absence or presence of LPS (10 μg/mL) for 6 h (Bar = 20 μm, *n* = 3). One-way ANOVA, Dunnett's post-hoc test. Data are presented as the mean ± SEM. ^*^P < 0.05, ^**^P < 0.01, ^***^P < 0.001 and ^****^P < 0.0001

The effect of SFN on autophagy in LPS-induced GMECs was further determined. As shown in Fig. 4A and B, pretreatment with SFN before the LPS challenge further increased bright blue dot staining of autophagic vacuoles compared to treatment with SFN or LPS alone (*P* < 0.5). Transmission electron microscopy revealed that autophagosome was observed in SFN-treated GMECs, which was further potentiated in LPS-induced GMECs after SFN pretreatment (Fig. 4C). Similarly, the upregulation of LC3-II expression was enhanced with SFN pretreatment under LPS stimulation (Fig. 4D and E, *P* < 0.001). To verify whether SFN promotes autophagic flux upon LPS-induced inflammation, GMECs were treated with Baf-A1, a late-stage autophagy inhibitor that potently blocks autophagosome-lysosome fusion. The results showed that combined treatment of Baf-A1 further enhanced the amplitude of LC3 puncta (Fig. 4F). These findings highlight that SFN-activated autophagy plays a vital role in LPS-induced GMECs.

### Nrf2 knockdown inhibits autophagy and attenuates antioxidant and anti-inflammatory activities of SFN in LPS-induced primary GMECs

**Fig. 5:**
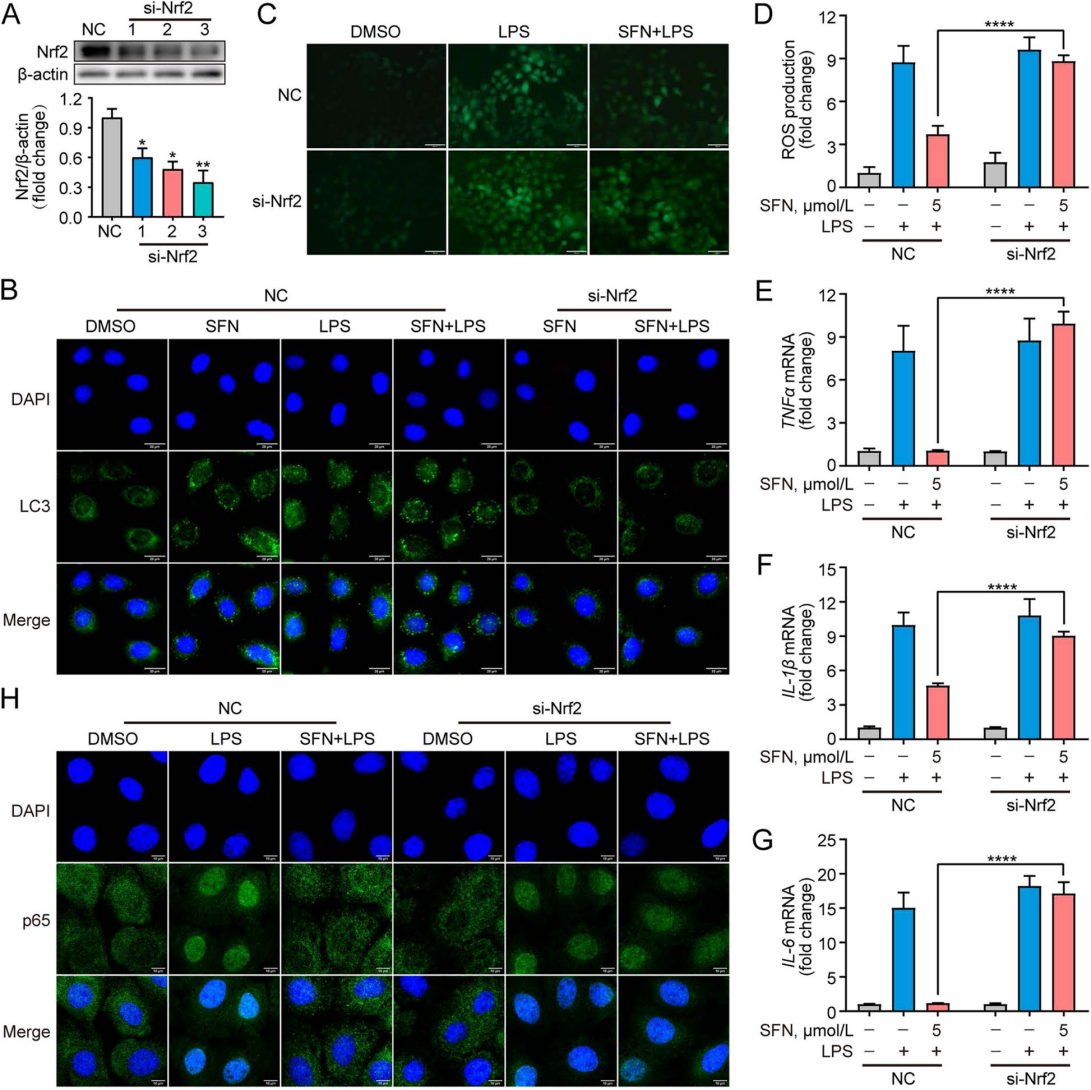
Nrf2 knockdown inhibited autophagy, attenuated antioxidant, anti-inflammatory activities of SFN in LPS-induced primary GMECs. Cells were transfected with si-Nrf2 for 48 h in advance and then treated with SFN (5 μmol/L) for 12 h in the absence or presence of LPS (10 μg/mL) for 6 h. **A** Western blot analysis of Nrf2 protein (*n* = 3). Cells were transfected with negative control (NC) and siRNA against Nrf2 (si-Nrf2) for 48 h. One-way ANOVA, Dunnett's post-hoc test. **B** Immunofluorescent analysis of LC3 puncta (green) and nuclear staining with DAPI (blue) (Bar = 20 μm, *n* = 3). **C** and **D** Analysis of ROS production (Bar = 100 μm). Two-way ANOVA, Tukey's post-hoc test. **E–G** qRT-PCR analysis of relative mRNA levels of inflammatory factors (*n* = 3). Two-way ANOVA, Tukey's post-hoc test. **H** Immunofluorescent analysis of NF-κBp65 and nuclear staining with DAPI (blue) (Bar = 10 μm). Data are presented as the mean ± SEM. ^*^P < 0.05, ^**^P < 0.01, ^***^P < 0.001 and ^****^P < 0.0001

Given the role of Nrf2 in regulating autophagy, oxidative stress, and inflammatory response [39], Nrf2-targeted strategies are important for the prevention of mastitis. Thus, we first used Nrf2-silenced GMECs to confirm the potential impact of SFN-activated Nrf2 signaling on the autophagy pathway. As shown in Fig. 5A, transfection with siRNA targeting Nrf2 decreased the protein level of Nrf2 in GMECs compared to the control group, with the most significant effect of si-Nrf2-3 (*P* < 0.01). We thus choose si-Nrf2-3 for subsequent experiments. Immunofluorescence staining showed that SFN enhanced the amplitude of LC3 puncta in LPS-induced GMECs, which however was decreased by knockdown of Nrf2 (Fig. 5B). To be of antioxidant and anti-inflammatory functional relevance, Nrf2 knockdown reverted SFN-mediated the decrease of ROS production and its inhibition of proinflammatory cytokine levels in LPS-induced GMECs (Fig. 5C–F, *P* < 0.0001). Consistently, using a siRNA targeting Nrf2 reverted SFN-induced reduction of NF-κBp65 nuclear translocation in LPS-induced GMECs (Fig. 5G). These results demonstrate that SFN-induced autophagy is dependent on the elevated Nrf2 level, which substantially contributes to improving the activities of antioxidant and anti-inflammation.

### SFN suppresses LPS-induced expression of inflammatory factors Nrf2 and LC3 in mouse mammary glands

**Fig. 6:**
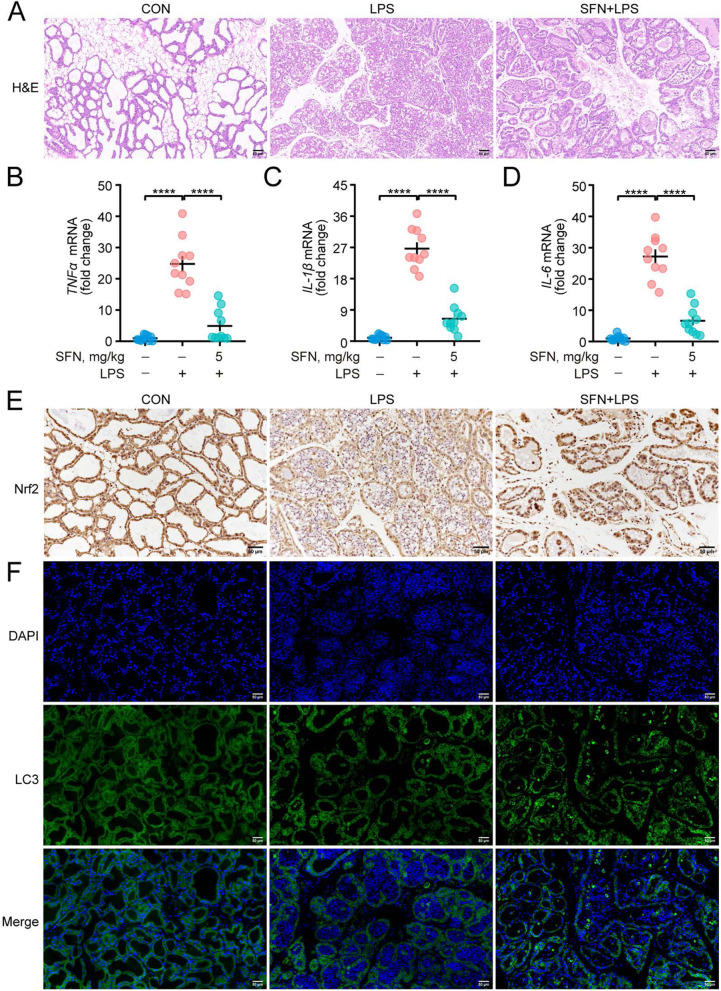
SFN suppressed the expression of inflammatory factors Nrf2 and LC3 in mouse mammary glands following LPS treatment. BALB/c mice were administered vehicle (saline) or SFN (50 mg/kg, i.p.) on days 4 to 10 of lactation. On day 9 of lactation, mice were infused with 100 μL LPS (0.2 mg/mL) into the fourth mammary glands through the mammary ducts. After 24 h, mammary glands were collected from 10 mice for each group. **A** Representative images of mammary gland stained with H&E in mice (Bar = 50 μm). **B–D** qRT-PCR analysis of mammary glands specimen from 10 mice for each group to determine expression of inflammatory factors (*n* = 10). **E** Immunohistochemistry staining of Nrf2 is shown (Bar = 50 μm). **F** Immunofluorescence staining of LC3 puncta (Bar = 50 μm). One-way ANOVA, Dunnett's post-hoc test. Data are presented as the mean ± SEM. ^*^P < 0.05, ^**^P < 0.01, ^***^P < 0.001 and ^****^P < 0.0001

The results shown above were further evaluated in an in vivo study to assess the potential effect of SFN on alleviating mastitis in the LPS-induced mice mastitis model. H&E staining was performed to examine histopathological changes in the mammary gland and the results showed that LPS stimulation resulted in a large number of neutrophils infiltrating the mammary acinar cavity, whereas SFN significantly inhibited this effect (Fig. 6A). Additionally, SFN decreased LPS-induced pro-inflammatory factors (*TNF-α*, *IL-1β*, and *IL-6*) mRNA expression in the mammary gland (Fig. 6B–D, *P* < 0.0001). As shown in Fig. 6E and F, SFN enhanced immunohistochemistry staining of Nrf2 and amplitude of LC3 puncta in the LPS-induced mammary gland. These results highlight that SFN pretreatment activates Nrf2 and autophagy and thereby prevents LPS-induced mastitis in mice.

## Discussion

Gram-negative bacteria, especially *Escherichia coli* (*E. coli*), are common pathogens of mastitis in ruminants [40–42]. LPS released from the bacterial cell wall causes a series of cellular immune responses, activating of inflammatory signaling, and producing of cytokines, and ROS [43]. Excessive ROS can further exacerbate the inflammatory response, which further increases the intensity of oxidative stress, leading to a vicious cycle of oxidative stress and inflammatory response [44]. Therefore, agents with anti-oxidative and anti-inflammatory activities may be beneficial in combating mastitis. In this study, primary dairy goat mammary epithelial cells and a mouse model of mastitis were used to evaluate the effect of SFN on LPS-induced inflammation. Results indicated that SFN inhibited inflammatory factors (*TNF-α*, *IL-1β*, and *IL-6*), inflammatory mediators (COX2 and iNOS) expression and NF-κB activation in LPS-induced GMECs. SFN exerted an antioxidant effect by activating Nrf2, up-regulating the expression of antioxidant enzymes, and reducing LPS-induced ROS generation in GMECs. Moreover, SFN effectively suppressed LPS-induced mastitis in mice. Mechanistically, these effects were largely attributed to Nrf2-mediated autophagy.

Mammary epithelial cells are the first line of defense against microbial invasion of the mammary gland [45]. Following an LPS challenge, the inflammatory factors are released from cells via activating the NF-κB pathways to regulate further infection [46]. Therefore, the anti-inflammatory activity of SFN was evaluated in LPS-induced primary GMECs. SFN pretreatment inhibited LPS-induced expression of inflammatory factors (*TNF-α*, *IL-1β*, and *IL-6*) mRNA levels and the inflammatory mediators (COX2 and iNOS) protein expression, which are closely involved in NF-κB. NF-κB plays a crucial role in the inflammatory response, governing the release of cytokines [47]. Previous studies revealed that NF-κB activation is regulated by IκBα phosphorylation and degradation [48]. Similarly, SFN reduced the levels of phosphor-IκBα and phosphor-NF-κBp65, as well as the nuclear translocation of NF-κBp65. Inflammatory response and its constant companion oxidative stress are key features and major mediators of mastitis initiation and progression. Excessive ROS accumulation is linked to oxidative stress and inflammation exaggeration. Accordingly, inhibiting the overproduction of ROS is a general approach to suppress inflammatory disease such as mastitis. Nrf2 has been identified as one of the key regulators of antioxidant responses, activating the transcription of several antioxidant genes that serve to counteract the effects of ROS. Wang et al. [49] demonstrated that aucubin can provide a neuroprotective effect against oxidative stress and inflammation by activating the Nrf2 pathway in traumatic brain injury. Zhang et al. [50] further suggested that rutaecarpine attenuates dextran sulfate sodium-induced colitis through the activation of Nrf2 signaling. In this study, the activation of Nrf2 was shown to up-regulate the expression of downstream antioxidant enzymes (HO-1, NQO1, GCLC, and GCLM) in LPS-induced GMECs, thereby scavenging ROS and enhancing the antioxidant capacity of SFN in LPS-induced GMECs. Altogether, the results suggest that the potential anti-inflammatory and anti-oxidative stress activities of SFN may be linked to Nrf2 activation in response to LPS stimulation.

To explore the potential mechanism of SFN in mitigating LPS-induced inflammation, additional experiments were conducted. Several studies have widely reported the involvement of autophagy in infection, immunity, and inflammation [18–20]. Cao et al. [51] found that autophagy was capable of mediating the inflammatory effects of punicalagin. Rong et al. [52] demonstrated that citrus peel flavonoid nobiletin enhanced autophagy to attenuate LPS-induced inflammation. Wang et al. [53] revealed that taurine alleviates *Streptococcus uberis*-induced inflammation by activating autophagy in mammary epithelial cells. Therefore, it was hypothesized that SFN alleviates LPS-induced inflammation by regulating autophagy. Correspondingly, the pretreatment of SFN in the presence of LPS further promoted autophagy compared to treatment with SFN or LPS treatment alone, which was more pronounced in the presence of the Baf-A1 treatment. In the autophagy pathway, ULK1 has been proposed to be phosphorylated by AMPK at S556 (autophagy activation) or by mTOR at S757 (autophagy inhibition) to regulate autophagy in mammalian cells [54–56]. Zhang et al. [57] revealed that the AMPK-ULK1-autophagy axis can be activated by negative pressure to promote osteoblast differentiation of rat mesenchymal stem cells and bone regeneration. Consistently, SFN treatment activated autophagy through the AMPK-ULK1 pathway, but not the mTOR signaling. Collectively, these results indicate that autophagy plays an important role in attenuating LPS-induced inflammation in SFN-treated GMECs.

In addition to coordinating oxidative responses, immunity, and inflammation, the Nrf2 transcription factor also plays a prominent role in regulating autophagy. Pajares et al. [58] found that Nrf2 regulates chaperone-mediated autophagy by regulating LAMP2A. A recent study demonstrated that the enhancement of the autophagy pathway is largely dependent on Nrf2, which contributes to improving anti-bacterial innate immunity and inflammation [39]. Therefore, targeted regulation of Nrf2 to mediate autophagy may affect therapeutic strategies for mastitis. In the study using a siRNA targeting Nrf2, the attenuated amplitude of LC3 puncta suggested that Nrf2 plays a significant role in promoting autophagy when treated with SFN under LPS stimulation. Moreover, SFN-mediated repression of ROS generation and inflammatory factor levels induced by LPS was abolished in Nrf2-silenced GMECs, suggesting that SFN exerts antioxidant and anti-inflammatory effects through Nrf2 signaling. To provide additional support, SFN was found to effectively alleviate LPS-induced mastitis in mice. This was accompanied by an enhancement of IHC staining of Nrf2 and amplitude of LC3 puncta in the mammary gland, also a result consistent with our findings in GMECs. Similar to previous studies [39, 59], our data confirm that SFN exerts antioxidant and anti-inflammatory functions by promoting Nrf2-mediated autophagy, which may improve prevention strategies for mastitis in dairy goats.

## Conclusions

In conclusion, our findings demonstrate that SFN ameliorated LPS-induced inflammation via inhibiting LPS-induced oxidative stress and inflammatory responses in primary GMECs and alleviating LPS-induced mastitis in mice. The mechanisms were associated with the activation of the Nrf2-mediated autophagic pathway. These results provide insights into mastitis development and suggest that SFN may be a potential prevention strategy for mastitis in dairy goats. Nevertheless, further studies are needed to elucidate the clinical application of SFN in dairy animals.

## Data Availability

All data generated or analyzed during this study are included in this published article.

## Abbreviations

AMPK: 5’-Adenosine monophosphate (AMP)-activated protein kinase
BafA1: Bafilomycin A1
COX2: Cyclooxygenase-2
CQ: Chloroquine
CBP: CREB binding protein
DAPI: 4′,6-Diamidino-2-phenylindole dihydrochloride
*E. coli*: *Escherichia coli*
GCLC: Glutamate-cysteine ligase catalytic
GCLM: Glutamate cysteine ligase modifier subunit
GMECs: Goat mammary epithelial cells
H&E: Hematoxylin–eosin
HO-1: Heme oxygenase-1
IHC: Immunohistochemistry
IL: Interleukin
iNOS: Inducible nitric oxide synthase
IκBα: Inhibitor kappa B α
LC3-II: LC3-phosphatidylethanolamine conjugate
LPS: Lipopolysaccharide
mTOR: Mechanistic target of rapamycin
NC: Negative control
NF-κB: Nuclear factor kappa-B
NQO1: NADPH quinone oxidoreductase 1
Nrf2: Nuclear factor erythroid 2-related factor 2
qPCR: Real-time quantitative PCR
ROS: Reactive oxygen species
SFN: Sulforaphane
siRNA: Small interfering RNA
S6: Ribosomal protein S6
TFEB: Transcription factor EB
TNF-α: Tumor necrosis factor α
ULK1: UNC-51-like kinase

## References

[1] Chen J, Xu J, Li J, Du L, Chen T, Liu P (2015). Epigallocatechin-3-gallate attenuates lipopolysaccharide-induced mastitis in rats via suppressing MAPK mediated inflammatory responses and oxidative stress. Int Immunopharmacol.

[2] Hu X, Guo J, Zhao C, Jiang P, Maimai T, Yanyi L (2020). The gut microbiota contributes to the development of Staphylococcus aureus-induced mastitis in mice. ISME J.

[3] Bourgonje AR, Feelisch M, Faber KN, Pasch A, Dijkstra G, Goor HV (2020). Oxidative stress and redox-modulating therapeutics in inflammatory bowel disease. Trends Mol Med.

[4] Matyas C, Haskó G, Liaudet L, Trojnar E, Pacher P (2021). Interplay of cardiovascular mediators, oxidative stress and inflammation in liver disease and its complications. Nat Rev Cardiol.

[5] Wu H, Wang Y, Zhang Y, Xu F, Chen J, Duan L (2020). Breaking the vicious loop between inflammation, oxidative stress and coagulation, a novel anti-thrombus insight of nattokinase by inhibiting LPS-induced inflammation and oxidative stress. Redox Biol..

[6] Wang ZW, Chen ZL, Jiang ZY, Luo P, Liu L, Huang Y (2019). Cordycepin prevents radiation ulcer by inhibiting cell senescence via NRF2 and AMPK in rodents. Nat Commun.

[7] Ma YF, Wu ZH, Gao M, Loor JJ (2018). Nuclear factor erythroid 2-related factor 2 antioxidant response element pathways protect bovine mammary epithelial cells against H_2_O_2_-induced oxidative damage in vitro. J Dairy Sci.

[8] Lu MC, Zhao J, Liu YT, Liu T, Tao MM, You QD (2019). CPUY192018, a potent inhibitor of the Keap1-Nrf2 protein-protein interaction, alleviates renal inflammation in mice by restricting oxidative stress and NF-kappaB activation. Redox Biol..

[9] Hu R, Saw CL, Yu R, Kong AT (2010). Regulation of NF-E2-related factor 2 signaling for cancer chemoprevention antioxidant coupled with antiinflammatory. Antioxid Redox Signal.

[10] Liao S, Wu J, Liu R, Wang S, Luo J, Yang Y (2020). A novel compound DBZ ameliorates neuroinflammation in LPS-stimulated microglia and ischemic stroke rats: Role of Akt(Ser473)/GSK3beta(Ser9)-mediated Nrf2 activation. Redox Biol..

[11] Jiang T, Tian F, Zheng H, Whitman SA, Lin Y, Zhang Z (2014). Nrf2 suppresses lupus nephritis through inhibition of oxidative injury and the NF-kappaB-mediated inflammatory response. Kidney Int.

[12] Ran X, Yan Z, Yang Y, Hu G, Liu J, Hou S (2020). Dioscin improves pyroptosis in LPS-induced mice mastitis by activating AMPK/Nrf2 and inhibiting the NF-kappaB signaling pathway. Oxid Med Cell Longev.

[13] Bellezza I, Mierla AL, Minelli A (2010). Nrf2 and NF-kappaB and their concerted modulation in cancer pathogenesis and progression. Cancers (Basel).

[14] Sivandzade F, Prasad S, Bhalerao A, Cucullo L (2019). NRF2 and NF-қB interplay in cerebrovascular and neurodegenerative disorders: molecular mechanisms and possible therapeutic approaches. Redox Biol..

[15] Guo W, Liu J, Li W, Ma H, Gong Q, Kan X (2020). Niacin alleviates dairy cow mastitis by regulating the GPR109A/AMPK/NRF2 signaling pathway. Int J Mol Sci.

[16] Guo W, Li W, Su Y, Liu S, Kan X, Ran X (2021). GPR109A alleviate mastitis and enhances the blood milk barrier by activating AMPK/Nrf2 and autophagy. Int J Biol Sci.

[17] Levine B, Klionsky DJ (2004). Development by self-digestion: review molecular mechanisms and biological functions of autophagy. Dev Cell.

[18] Deretic V, Levine B (2018). Autophagy balances inflammation in innate immunity. Autophagy.

[19] Levine B, Mizushima N, Virgin HW (2011). Autophagy in immunity and inflammation. Nature.

[20] Deretic V (2021). Autophagy in inflammation, infection, and immunometabolism. Immunity.

[21] Sureshbabu A, Ryter SW, Choi ME (2015). Oxidative stress and autophagy: crucial modulators of kidney injury. Redox Biol.

[22] Sun X, Chang R, Tang Y, Luo S, Jiang C, Jia H (2021). Transcription factor EB (TFEB)-mediated autophagy protects bovine mammary epithelial cells against H_2_O_2_-induced oxidative damage in vitro. J Anim Sci Biotechnol.

[23] Guo WJ, Liu JX, Zhang YF, Ma H, Li YH, Gong Q (2020). Dehydroandrographolide inhibits mastitis by activating autophagy without affecting intestinal flora. Aging-US.

[24] Jo C, Gundemir S, Pritchard S, Jin YN, Rahman I, Johnson GV (2014). Nrf2 reduces levels of phosphorylated tau protein by inducing autophagy adaptor protein NDP52. Nat Commun.

[25] Pajares M, Jimenez-Moreno N, Garcia-Yague AJ, Escoll M, Ceballos ML, Leuven FV (2016). Transcription factor NFE2L2/NRF2 is a regulator of macroautophagy genes. Autophagy.

[26] Jain A, Lamark T, Sjottem E, Larsen KB, Awuh JA, Overvatn A (2010). p62/SQSTM1 is a target gene for transcription factor NRF2 and creates a positive feedback loop by inducing antioxidant response element-driven gene transcription. J Biol Chem.

[27] Kapuy O, Papp D, Vellai T, Banhegyi G, Korcsmáros T (2018). Systems-level feedbacks of NRF2 controlling autophagy upon oxidative stress response. Antioxidants (Basel)..

[28] Russo M, Spagnuolo C, Russo GL, Skalicka-Wozniak K, Daglia M, Sobarzo-Sanchez E (2018). Nrf2 targeting by sulforaphane: A potential therapy for cancer treatment. Crit Rev Food Sci Nutr.

[29] Pan MH, Lai CS, Dushenkov S, Ho CT (2009). Modulation of inflammatory genes by natural dietary bioactive compounds. J Agric Food Chem.

[30] Tasneem S, Liu B, Li B, Choudhary MI, Wang W (2019). Molecular pharmacology of inflammation: medicinal plants as anti-inflammatory agents. Pharmacol Res.

[31] Okada M, Yamamoto A, Aizawa SI, Taga A, Terashima H, Kodama S (2017). HPLC separation of sulforaphane enantiomers in broccoli and its sprouts by transformation into diastereoisomers using derivatization with (S)-Leucine. J Agric Food Chem..

[32] Liu H, Talalay P (2013). Relevance of anti-inflammatory and antioxidant activities of exemestane and synergism with sulforaphane for disease prevention. Proc Natl Acad Sci U S A.

[33] Liu H, Yang X, Tang K, Ye T, Duan C, Lv P (2020). Sulforaphane elicts dual therapeutic effects on renal inflammatory Injury and crystal deposition in calcium oxalate nephrocalcinosis. Theranostics.

[34] Xin Y, Bai Y, Jiang X, Zhou S, Wang Y, Wintergerst KA (2018). Sulforaphane prevents angiotensin II-induced cardiomyopathy by activation of Nrf2 via stimulating the Akt/GSK-3ss/Fyn pathway. Redox Biol.

[35] Wang JJ, Wei ZK, Zhang X, Wang YN, Fu YH, Yang ZT (2017). Butyrate protects against disruption of the blood-milk barrier and moderates inflammatory responses in a model of mastitis induced by lipopolysaccharide. Br J Pharmacol.

[36] Bulgari O, Dong X, Roca AL, Caroli AM, Loor JJ (2017). Innate immune responses induced by lipopolysaccharide and lipoteichoic acid in primary goat mammary epithelial cells. J Anim Sci Biotechnol.

[37] Zhang H, Li C, Wen D, Li R, Lu S, Xu R (2022). Melatonin improves the quality of maternally aged oocytes by maintaining intercellular communication and antioxidant metabolite supply. Redox Biol..

[38] Ahmed SMU, Luo L, Namani A, Wang XJ, Tang XW (2017). Nrf2 signaling pathway: pivotal roles in inflammation. Biochim Biophys Acta Mol Basis Dis.

[39] Zhang W, Cheng C, Sha Z, Chen C, Yu C, Lv N (2021). Rosmarinic acid prevents refractory bacterial pneumonia through regulating Keap1/Nrf2-mediated autophagic pathway and mitochondrial oxidative stress. Free Radic Biol Med.

[40] Wang YM, Ma YQ, Bi SC, Ma XD, Guan R, Wang SH (2019). Therapeutic effect of ginsenoside Rg1 on mastitis experimentally induced by lipopolysaccharide in lactating goats. J Dairy Sci.

[41] Yang G, Yue Y, Li D, Duan C, Qiu X, Zou Y (2020). Antibacterial and immunomodulatory effects of pheromonicin-NM on *Escherichia coli*-challenged bovine mammary epithelial cells. Int Immunopharmacol..

[42] Wu Q, Liu MC, Yang J, Wang JF, Zhu YH (2016). *Lactobacillus **rhamnosus* GR-1 ameliorates *Escherichia coli*-induced inflammation and cell damage via attenuation of ASC-independent NLRP3 inflammasome activation. Appl Environ Microbiol.

[43] Fang M, Zou T, Yang X, Zhang Z, Cao P, Han J (2021). Discovery of novel pterostilbene derivatives that might treat sepsis by attenuating oxidative stress and inflammation through modulation of MAPKs/NF-kappaB signaling pathways. Antioxidants (Basel)..

[44] Xu T, Hu S, Liu Y, Sun K, Luo L, Zeng L (2022). Hawk tea flavonoids as natural hepatoprotective agents alleviate acute liver damage by reshaping the intestinal microbiota and modulating the Nrf2 and NF-kappaB signaling pathways. Nutrients..

[45] Swanson KM, Stelwagen K, Dobson J, Henderson HV, Davis SR, Farr VC (2009). Transcriptome profiling of *Streptococcus **uberis*-induced mastitis reveals fundamental differences between immune gene expression in the mammary gland and in a primary cell culture model. J Dairy Sci.

[46] Shembade N, Harhaj NS, Liebl DJ, Harhaj EW (2007). Essential role for TAX1BP1 in the termination of TNF-alpha-, IL-1- and LPS-mediated NF-kappaB and JNK signaling. EMBO J.

[47] Iliopoulos D, Hirsch HA, Struhl K (2009). An epigenetic switch involving NF-kappaB, Lin28, Let-7 microRNA, and IL6 links inflammation to cell transformation. Cell.

[48] Hayden MS, Ghosh S (2008). Shared principles in NF-kappaB signaling. Cell.

[49] Wang H, Zhou X, Wu L, Liu G, Xu W, Zhang X (2020). Aucubin alleviates oxidative stress and inflammation via Nrf2-mediated signaling activity in experimental traumatic brain injury. J Neuroinflammation..

[50] Zhang Y, Yan T, Sun D, Xie C, Wang T, Liu X (2020). Rutaecarpine inhibits KEAP1-NRF2 interaction to activate NRF2 and ameliorate dextran sulfate sodium-induced colitis. Free Radic Biol Med.

[51] Cao Y, Chen J, Ren G, Zhang Y, Tan X, Yang LN (2019). Punicalagin prevents inflammation in LPS-induced RAW264.7 macrophages by inhibiting FoxO3a/autophagy signaling pathway. Nutrients..

[52] Rong X, Xu J, Jiang Y, Li F, Chen Y, Dou QP (2021). Citrus peel flavonoid nobiletin alleviates lipopolysaccharide-induced inflammation by activating IL-6/STAT3/FOXO3a-mediated autophagy. Food Funct.

[53] Wang Z, Lan R, Xu Y, Zuo J, Han X, Phouthapane V, et al. Taurine alleviates *Streptococcus uberis*-induced inflammation by activating autophagy in mammary epithelial cells. Front Immunol. 2021;12:631113. 10.3389/fimmu.2021.631113. 10.3389/fimmu.2021.631113 PMC7996097 33777017

[54] Fan Y, Wang N, Rocchi A, Zhang W, Vassar R, Zhou Y (2017). Identification of natural products with neuronal and metabolic benefits through autophagy induction. Autophagy.

[55] Li GM, Li L, Li MQ, Chen X, Su Q, Deng ZJ (2021). DAPK3 inhibits gastric cancer progression via activation of ULK1-dependent autophagy. Cell Death Differ.

[56] Lee DH, Park JS, Lee YS, Han J, Lee DK, Kwon SW (2020). SQSTM1/p62 activates NFE2L2/NRF2 via ULK1-mediated autophagic KEAP1 degradation and protects mouse liver from lipotoxicity. Autophagy.

[57] Zhang S, Xie Y, Yan F, Zhang Y, Yang Z, Chen Z (2022). Negative pressure wound therapy improves bone regeneration by promoting osteogenic differentiation via the AMPK-ULK1-autophagy axis. Autophagy.

[58] Pajares M, Rojo AI, Arias E, Diaz-Carretero A, Cuervo AM, Cuadrado A (2018). Transcription factor NFE2L2/NRF2 modulates chaperone-mediated autophagy through the regulation of LAMP2A. Autophagy.

[59] Chang R, Sun X, Jia H, Xu Q, Dong Z, Tang Y (2022). Inhibiting nuclear factor erythroid 2 related factor 2-mediated autophagy in bovine mammary epithelial cells induces oxidative stress in response to exogenous fatty acids. J Anim Sci Biotechnol.

